# Perforin-Like Proteins of Apicomplexan Parasites

**DOI:** 10.3389/fcimb.2020.578883

**Published:** 2020-09-15

**Authors:** Juliane Sassmannshausen, Gabriele Pradel, Sandra Bennink

**Affiliations:** Division of Cellular and Applied Infection Biology, Institute of Zoology, Rheinisch-Westfälische Technische Hochschule Aachen University, Aachen, Germany

**Keywords:** apicomplexa, *Plasmodium falciparum*, malaria, *Toxoplasma gondii*, perforin, MACPF domain, host cell egress, cell traversal

## Abstract

Perforins are secreted proteins of eukaryotes, which possess a membrane attack complex/perforin (MACPF) domain enabling them to form pores in the membranes of target cells. In higher eukaryotes, they are assigned to immune defense mechanisms required to kill invading microbes or infected cells. Perforin-like proteins (PLPs) are also found in apicomplexan parasites. Here they play diverse roles during lifecycle progression of the intracellularly replicating protozoans. The apicomplexan PLPs are best studied in *Plasmodium* and *Toxoplasma*, the causative agents of malaria and toxoplasmosis, respectively. The PLPs are expressed in the different lifecycle stages of the pathogens and can target and lyse a variety of cell membranes of the invertebrate and mammalian hosts. The PLPs thereby either function in host cell destruction during exit or in overcoming epithelial barriers during tissue passage. In this review, we summarize the various PLPs known for apicomplexan parasites and highlight their roles in *Plasmodium* and *Toxoplasma* lifecycle progression.

## Introduction

Members of the pore-forming Membrane Attack Complex/Perforin (MACPF) superfamily are highly conserved in both prokaryotes and eukaryotes, and are mainly used for immune defense or virulence. During the co-evolution of pathogens and hosts, both have developed pore-forming proteins facilitating target membrane lysis and translocation of molecules.

Eukaryotic parasites of the phylum Apicomplexa express MACPF domain-containing proteins termed perforin-like proteins (PLPs). The Apicomplexa comprises a diverse group of intracellularly replicating protozoans that share the apical complex, composed of secretory organelles, such as the micronemes and rhoptries—and structural elements. Calcium-regulated protein discharge from the micronemes is fundamental to motility, cell invasion and egress of apicomplexan parasites (e.g., reviewed in Blackman and Bannister, 2001; Dubois and Soldati-Favre, 2019). Members of the phylum Apicomplexa include parasites that can cause infectious diseases relevant to human or veterinary medicine, such as the malaria parasite *Plasmodium* or representatives of the genera *Toxoplasma, Babesia*, and *Eimeria*.

Many apicomplexan parasites exhibit complex lifecycles, during which they reproduce both asexually and sexually, and which include one or more hosts. For example, the malaria parasite *Plasmodium* is transmitted from human to human via the bite of an *Anopheles* mosquito, which serves as the definitive host. The parasite *Toxoplasma gondii* on the other hand infects members of the family Felidae (domestic cats and their relatives) as its definitive host, but can use other vertebrates as intermediate hosts. Lifecycle progression of apicomplexan parasites is highly dependent on host cell traversal as well as invasion of and egress from host cells.

We here review the role of apicomplexan PLPs (ApiPLPs) during lifecycle progression, with special focus on the processes of cell traversal and host cell egress. As most work about ApiPLPs has been done on *Plasmodium* and *T. gondii*, we will concentrate on these species as representatives.

## Conservation of Apicomplexan MACPF Proteins

The MACPF superfamily is named after the central protein domain shared by the membrane attack complex (MAC) proteins of the human complement system (C6, C7, C8α, C8β, and C9) and the cytolytic perforin (PF) of cytotoxic T lymphocytes and natural killer cells. However, members of the superfamily can be found in all three domains of life, i.e., eubacteria, archaebacteria, and eukaryotes (Moreno-Hagelsieb et al., 2017). In eukaryotes, they are not only involved in immune defense mechanisms, but additionally play important roles in various biological processes, such as embryonic development or neural migration (reviewed in Lukoyanova et al., 2016).

Among the phylum of Apicomplexa, which almost exclusively comprises obligatory intracellular parasites, MACPF domain-containing proteins are encoded in all genomes sequenced so far, except for *Cryptosporidium*, which might have lost the genes during evolution. The number of MACPF proteins however varies between the different species, which might be linked to the degree of lifecycle complexity (see Table 1). In general, parasites that are transmitted to vertebrates via insects, such as *Plasmodium, Theileria*, or *Babesia*, seem to express more PLPs than parasites, which are restricted to vertebrates, e.g., *Toxoplasma, Neospora*, or *Eimeria*. While *Plasmodium* parasites express five and *Theileria* and *Babesia* six to nine PLPs, *Toxoplasma, Neospora*, and *Eimeria* only express two to three PLPs.

**Table 1:** Putative apicomplexan PLPs with their gene identification numbers according to EuPathDB.org (Aurrecoechea et al., 2017), molecular weight (MW), peak expression of the plasmodial genes (López-Barragán et al., 2011; Otto et al., 2014), and function.

**Organism**	**GeneID EuPathDB and protein name**	**MW (kDa)**	**Peak expression**	**Function**	**References**
*Plasmodium falciparum*	PF3D7_0408700 (PPLP1)	94	GC	Traversal of human cells (sporozoite); Host cell egress (merozoite)	Garg et al., 2013; Yang et al., 2017
	PF3D7_1216700 (PPLP2)	124	OK	Host cell egress (gametocyte)	Wirth et al., 2014; Hentzschel et al., 2020
	PF3D7_0923300 (PPLP3)	93	OK		
	PF3D7_0819400 (PPLP4)	76	OK	Traversal of mosquito midgut epithelium (ookinete)	Wirth et al., 2015
	PF3D7_0819200 (PPLP5)	79	OK		
*Plasmodium berghei*	PBANKA_1006300 (PPLP1)	90	OK	Traversal of sinusoidal endothelium (sporozoite)	Ishino et al., 2005
	PBANKA_1432400 (PPLP2)	114	GC	Host cell egress (gametocyte)	Deligianni et al., 2013
	PBANKA_0824200 (PPLP3)	92	GC, OK	Traversal of mosquito midgut epithelium (ookinete)	Kadota et al., 2004
	PBANKA_0711400 (PPLP4)	79	OK	Traversal of mosquito midgut epithelium (ookinete)	Deligianni et al., 2018
	PBANKA_0711600 (PPLP5)	80	OK	Traversal of mosquito midgut epithelium (ookinete)	Ecker et al., 2007
*Toxoplasma gondii*	TGME49_204130 (TgPLP1)	125		Vacuolar and host cell egress (tachyzoite)	Kafsack et al., 2009; Roiko and Carruthers, 2013; Guerra et al., 2018
	TGME49_272430 (TgPLP2)	92			
*Theileria annulata*	TA19210	126			
	TA11680	92			
	TA07905	57			
	TA07910	67			
	TA18285^a^	140			
	TA18325	39			
	TA14315	13			
*Babesia bovis*	BBOV_IV001370	108			
	BBOV_II007150	84			
	BBOV_III000410^a^	143			
	BBOV_II002020	46			
	BBOV_II001970	61			
	BBOV_III000320	56			
	BBOV_II006750	45			
*Neospora caninum*	NCLIV_020990	114			
	NCLIV_034870	95			
	NCLIV_035170	151			
*Eimeria tenella*	ETH_00005740	83			
	ETH_00025550	148			

*GC, gametocyte; OK, ookinete*.

a *Three MACPF domains predicted*.

## Domain Architecture and Mechanism of Pore Formation

**Figure 1:**
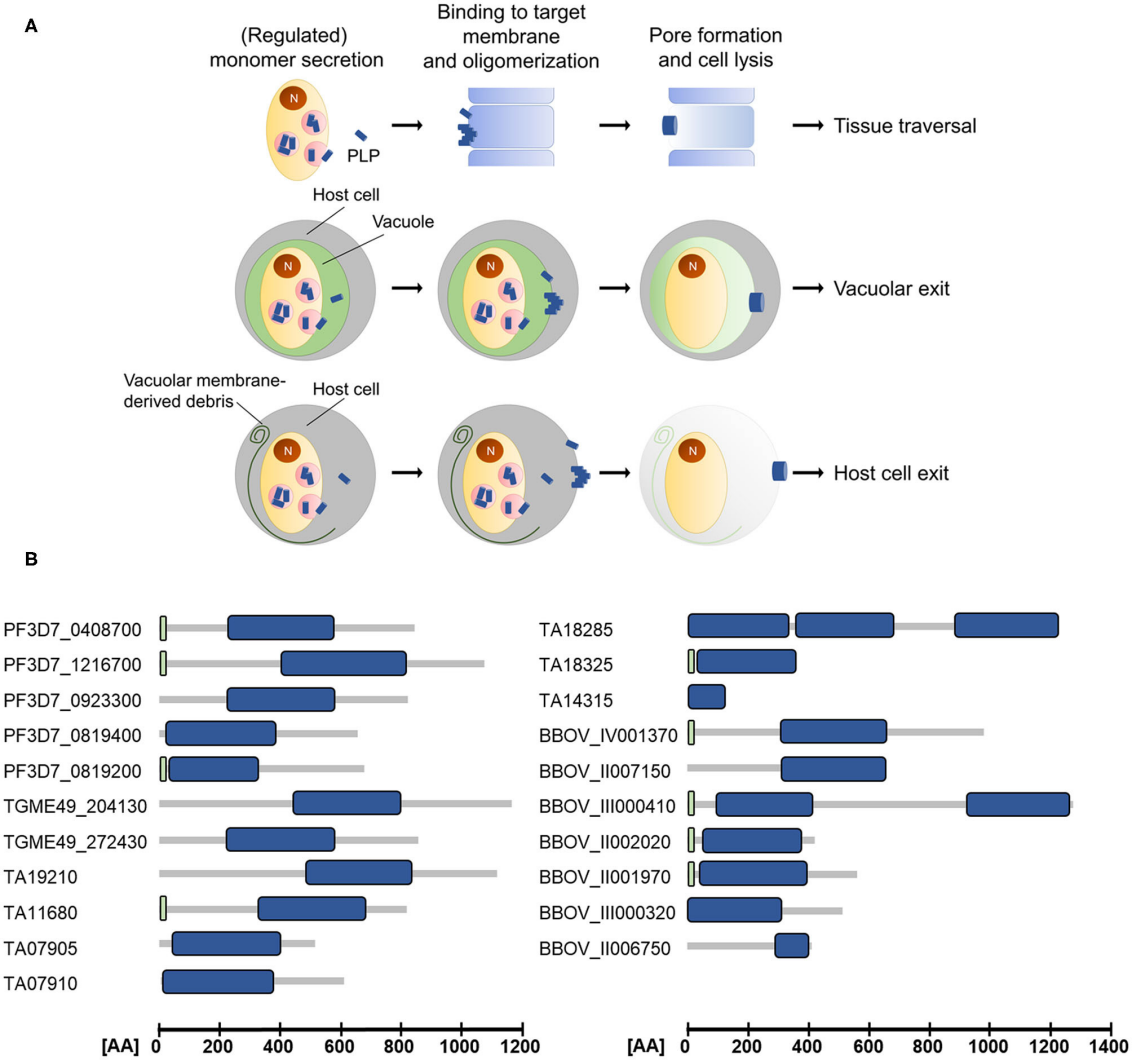
**(A)** Schematic depicting the modes of action of apicomplexan perforin-like proteins (PLPs). Target membrane lysis mediated by PLP activity is used for cell traversal, egress from the parasitophorous vacuole and the subsequent host cell exit. N, nucleus. **(B)** Domain architecture of selected ApiPLPs from *P. falciparum, T. gondii, T. annulata*, and *B. bovis*. Blue boxes indicate MACPF domains, green boxes indicate signal peptides (predicted using SignalP 5.0; Almagro Armenteros et al., 2019). AA, amino acids.

The apicomplexan PLPs characterized so far share the basic architecture of canonical MACPF proteins but additionally exhibit some unique features. They are composed of a central MACPF domain, surrounded by a variable N-terminal region and an apicomplexan-specific β-pleated sheet-rich C-terminal region (reviewed in Kafsack and Carruthers, 2010). The N-terminal region does not only vary in length and sequence between different apicomplexan PLPs (see Figure 1), but also between proteins of the same species. For example the two MACPF-domain containing proteins that are expressed in *T. gondii*, TgPLP1, and TgPLP2, only share 13% sequence identity in the N-terminal regions, while the C-terminal regions are 36% identical.

The canonical MACPF domain, which is structurally similar to prokaryotic cholesterol-dependent cytolysins (CDCs), consists of a central four-stranded β-sheet that is flanked by two α-helical clusters (Hadders et al., 2007; Rosado et al., 2007). The α-helical clusters display the typical pattern of alternating hydrophilic and hydrophobic amino acids and convert into amphipathic transmembrane β-hairpins during pore-formation (Shepard et al., 1998; Shatursky et al., 1999; reviewed in Tweten, 2005; Law et al., 2010; Aleshin et al., 2012). Interestingly, *Theileria* and *Babesia* are predicted to encode PLPs that comprise more than one MACPF domain (see Figure 1 and Table 1), which might enable pore-formation using a reduced number of monomers. Further, the MACPF domains of ApiPLPs exhibit several unique characteristics that are absent in canonical MACPF domains. They consist of a pair of anti-parallel α-helices and two pairs of cysteine residues, which are presumably responsible for stabilization. While the first pair is thought to stabilize the unique anti-parallel α-helices in the MACPF domain, the second one might support two strands of the central β-sheet (reviewed in Kafsack and Carruthers, 2010). The C-terminal regions of ApiPLPs contain a β-sheet-rich domain, called APC-β (ApiPLP C-terminal β-pleated sheet) domain, unique to the Apicomplexa, which is probably necessary for initial binding to the target membrane. Analyses of the *T. gondii* PLP TgPLP1 revealed that both, the N-terminal domain as well as the C-terminal domain, have membrane-binding activity, as shown in membrane flotation experiments with recombinant N- and C-terminal domains of the protein. However, Roiko and Carruthers (2013) demonstrated that only the C-terminal domain is critical for protein function. The authors generated several domain deletion strains expressing separate domains or domain combinations of the protein and subjected them to lysis assays, PVM permeabilization experiments and mouse virulence assays. In these experiments, parasites expressing the MACPF domain and C-terminal region of TgPLP1 mostly behaved like the wildtype, whereas parasites lacking one of these domains had no TgPLP1 activity (Roiko and Carruthers, 2013).

Recently published crystal structures of purified APC-β domains of TgPLP1 gave insight into the architecture and membrane-binding properties of ApiPLPs (Guerra et al., 2018; Ni et al., 2018). The studies revealed that the TgPLP1 APC-β domain has an unusual β-prism fold comprising three subdomains. One of these subdomains exhibits a protruding hydrophobic loop tipped by an exposed tryptophan that is presumably responsible for membrane insertion upon binding (Guerra et al., 2018; Ni et al., 2018). Accordingly, mutant parasites expressing TgPLP1 with a loop that is either shortened or changed in amino acid identity or hydrophobicity display an impaired egress phenotype recapitulating the TgPLP1-knock out phenotype, including the formation of smaller plaques compared to wildtype parasites, impaired PVM rupture and delayed egress as determined via LDH activity measurement in culture supernatants (see below; Kafsack et al., 2009; Guerra et al., 2018).

Successful pore formation by perforin-like proteins relies on several consecutive steps and begins with the release of soluble monomers that bind to their target membrane typically via their C-terminal domain. Oligomerization of PLP monomers by lateral interactions results in the formation of a ring-like structure, the so-called pre-pore that is not yet fully inserted into the membrane. Only after conformational rearrangement of the MACPF domain, during which the two α-helical clusters transform into transmembrane β-hairpins, a β-barrel pore is formed that finally inserts into the target membrane (Shepard et al., 1998, 2000; Shatursky et al., 1999; Law et al., 2010; Lukoyanova et al., 2015). MACPF pores are typically between 80 and 200 Å in diameter and contain 13-20 monomers (reviewed in Pipkin and Lieberman, 2007; Rosado et al., 2007; Lukoyanova et al., 2015). Although gel analyses of TgPLP1 complexes indicated the presence of more than 20 monomers comprising the pore complex (Roiko and Carruthers, 2013), recent crystallographic studies suggested that the TgPLP1 MACPF domain forms rather small, hexameric assemblies (Ni et al., 2018).

In order to ensure membrane specificity, the pore-forming process has to be tightly controlled. Mechanisms to avoid lysis of non-target membranes have been best studied for the pore-formation by human immune molecules, such as perforins and members of the complement system, but potentially also apply for the pore-formation by apicomplexan PLPs. These mechanisms might include the binding to an inhibitor protein prior to the lytic function of the PLP, regulated secretion, pH-dependent activity, protease-mediated activation or the interaction with specific phospholipids in the target membranes, as it has been shown for TgPLP1 (see below). Non-target membranes might further be protected from lysis through the presence of inhibitor proteins as it has been shown in humans as a mechanism to avoid self-cell destruction by the complement system (e.g., reviewed in Pipkin and Lieberman, 2007; Kafsack and Carruthers, 2010; Meri, 2016).

## Apicomplexan Perforins in Host Cell Egress

PLPs are involved in the exit of apicomplexan parasites from their respective host cells and hence have important roles for parasite propagation. Apicomplexan parasites mainly egress from their host cells by active lysis of the surrounding membranes, the parasitophorous vacuole membrane (PVM), and the host cell membrane (HCM). Host cell exit follows a strictly regulated programme, during which rupture of the PVM precedes HCM breakdown (the so-called inside-out egress). Both steps may involve lytic PLPs (Figure 1). The involvement of PLPs in host cell lysis, however, has so far only been shown experimentally for *Toxoplasma* and *Plasmodium* parasites (reviewed in Kafsack and Carruthers, 2010; Wirth and Pradel, 2012; Flieger et al., 2018).

Of the two PLPs detected in *T. gondii*, only TgPLP1 hitherto showed a clear involvement in host cell exit (Kafsack et al., 2009; Roiko and Carruthers, 2013; Guerra et al., 2018). TgPLP1 localizes to the micronemes of tachyzoites and is secreted in a calcium-dependent manner during egress similar to other micronemal proteins. Parasites lacking TgPLP1 remain enclosed by the PVM and the HCM, demonstrating its role in lysis of these membranes (Kafsack et al., 2009; Roiko and Carruthers, 2013). Before egress, cytosolic calcium concentrations are suppressed by the activity of a protein kinase A (PKA) and *T. gondii* tachyzoites impaired in PKA signaling spontaneously egress from their host cells in a TgPLP1-dependent fashion (Uboldi et al., 2018). However, it is not yet known if PKA directly regulates TgPLP1 secretion and activity or if they are part of two different pathways that are required for parasite egress.

It is postulated that the egress of *T. gondii* tachyzoites from the host cell is dependent on PV acidification, which promotes membrane binding of TgPLP1 (Roiko et al., 2014). TgPLP1 activity appears to be further regulated by interaction with specific phospholipids located in the respective membrane, particularly phosphatidylethanolamine (PE) or phosphatidylserine (PS), which are characteristic components of the inner leaflet of the red blood cell membrane. The availability of PE and PS, e.g., during egress, increases TgPLP1 activity, whereas the absence of these preferred phospholipid receptors in the outer leaflet of the target cell, e.g., during cell invasion, strongly limits the activity of TgPLP1 (Guerra et al., 2018). In a first model of tachyzoite egress, it has been postulated that TgPLP1 is transported to the PV after secretion, where it interacts with the PVM. This interaction triggers the lytic activity of the protein. After dissolution of the PVM, TgPLP1 binds to phospholipids of the HCM and, hence, further lyses this membrane, in consequence facilitating the final exit of the parasite from its host cell (Guerra et al., 2018).

Active host cell lysis by membrane breaching is also a typical mechanism used by the *Plasmodium* blood stages during exit from the red blood cell (RBC) (reviewed in Wirth and Pradel, 2012; Flieger et al., 2018). Two types of blood stages actively destroy the enveloping RBC during egress, the merozoites and the gametocytes, and for both stages, the involvement of plasmodial PLPs (PPLPs) has been reported. Like for *T. gondii*, egress of *Plasmodium* from the host cell is mediated by a signaling cascade that involves the sequential activation of a PKG by cGMP and of CDPKs by increased cytosolic calcium and this process results in the discharge of vesicles important for RBC lysis (reviewed in Flieger et al., 2018). While five PPLPs (termed PPLP1 to PPLP5) are encoded in the *Plasmodium* genome, only for PPLP1 and PPLP2, an involvement in RBC egress was hitherto shown (Kaiser et al., 2004; Deligianni et al., 2013; Garg et al., 2013; Wirth et al., 2014).

In *P. falciparum*, PPLP1 expression starts in the trophozoite stage and peaks in the mature schizont, where it initially localizes to the micronemes of the merozoites. Similar to TgPLP1, PPLP1 is secreted by the micronemes in a calcium-dependent fashion at the onset of RBC egress (Garg et al., 2013). Later, PPLP1 localizes to the PVM and RBC membrane (RBCM). Since recombinant PPLP1 demonstrates membrane-lytic activities, a role in membrane rupture during merozoite egress from the RBC was postulated. In this context, inhibition of the type-2 phosphatic acid phosphatase PAP2 of *P. falciparum* by propranolol results in the early microneme secretion of PPLP1 by merozoites and in consequence RBCM lysis (Kumar Sah et al., 2019). In general, PAP2s are able to phosphorylate diacylglycerol to generate phosphatidic acid, the latter of which was previously shown to be crucial for triggering microneme secretion in *T. gondii* tachyzoites (Bullen et al., 2016).

PPLP2 is involved in RBC lysis during egress of the *Plasmodium* gametocytes at the onset of gametogenesis (Deligianni et al., 2013; Wirth et al., 2014). In gametocytes, PPLP2 localizes to distinct vesicles which probably represent specialized egress vesicles presumably containing further egress-related molecules. Interestingly, these PPLP2-harboring vesicles are negative for the protein G377 which is a known component of osmiophilic bodies (OBs) (Wirth et al., 2014). OBs constitute vesicles of mature gametocytes that release their content into the PV lumen during the first minutes after gametocyte activation. They contain a variety of proteins, e.g., G377, MDV-1/Peg3, GEST, and several proteases, which are probably involved in PVM rupture (reviewed in Flieger et al., 2018). In a subsequent step, PPLP2 is discharged from the second type of vesicles in a calcium-dependent process. In accord with these data, PPLP2 was found to be a component of the *P. berghei* gametocyte egressome (Kehrer et al., 2016). In both *P. berghei* and *P. falciparum*, activated gametocytes deficient of PPLP2 remain trapped in the host RBCs (Deligianni et al., 2013; Wirth et al., 2014; Hentzschel et al., 2020). While the PVM ruptures normally, lack of PPLP2 leads to impaired perforation of the RBC membrane, which is essential to release the erythrocyte cytoplasm prior to the final rupture of the RBCM. In agreement with these data, recombinant PPLP2 was shown to form pores in RBCMs leading to hemoglobin release (Garg et al., 2020; this issue). The recombinant protein was further able to induce senescence in bystander RBCs. The lytic activity of recombinant PPLP2 could be blocked by specific MACPF domain inhibitors, suggesting that the plasmodial perforins may represent targets for future antimalarials.

## Apicomplexan Perforins in Tissue Traversal

The PPLPs of *Plasmodium* are further crucial for tissue traversal during lifecycle progression of the parasite (Figure 1). With the exception of PPLP2, all of the PPLPs were shown to be involved in crossing of epithelial barriers.

The passage through host cell epithelia is particularly important for the infective sporozoites during their journey to the human liver. An initial study on *P. berghei* demonstrated that PPLP1 (originally termed SPECT2) is present in sporozoite micronemes and secreted, when these traverse the sinusoidal endothelium (Ishino et al., 2005). Crossing of cells lining capillaries and the subsequent traversal of hepatocytes is a mandatory step of sporozoites, before these are able to settle down in a host hepatocyte to initiate replication (Mota et al., 2001, 2002; Pradel and Frevert, 2001; Amino et al., 2008; Tavares et al., 2013). *P. berghei* sporozoites deficient of PPLP1 remain in the blood circulation and are unable to establish an infection in mice (Ishino et al., 2005). Similarly, PPLP1-deficient *P. falciparum* sporozoites could not initiate an infection in the humanized mouse model (Yang et al., 2017). A subsequent study on the rodent malaria parasite *P. yoelii* showed that sporozoites traverse cell barriers via a transient vacuole, which is independent of moving junction formation, and that the sporozoites escape this vacuole with the help of PPLP1 (Risco-Castillo et al., 2015). The final invasion of hepatocytes requires a moving junction-dependent PV formation, in which the parasite in then able to grow.

Another epithelial crossing occurs during exit of the mosquito midgut by the motile *Plasmodium* ookinetes. In *P. berghei*, PPLP3 (originally termed MAOP), PPLP4 and PPLP5 were shown to be essential for the traversal of the mosquito midgut epithelium by ookinetes, while in *P. falciparum*, only PPLP4 has been attributed a role in this process so far (Kadota et al., 2004; Ecker et al., 2007; Wirth et al., 2015; Deligianni et al., 2018). Ookinetes lacking any of the three PPLPs are unable to infect female *Anopheles* mosquitoes. Interestingly, while in *P. falciparum*, PPLP4 is initially expressed in female gametocytes and later localizes to the ookinete micronemes, in *P. berghei*, PPLP4 was reported to be present on the entire surface of the ookinete (Wirth et al., 2015; Deligianni et al., 2018). While these data demonstrate a crucial role for PPLP3, PPLP4, and PPLP5 in the mosquito-specific lifecycle phase of the malaria parasite, the detailed mode of action and any potential synergistic interplay of the three perforins during mosquito midgut traversal still needs to be elucidated.

## Conclusion

Despite an increasing number of publications that shed light on the structure of apicomplexan PLPs and their functions during parasitic lifecycle progression, many questions about their mode of action and regulation remain. For instance, further studies are needed to determine how membrane specificity is achieved. What are the receptors for initial membrane binding and how are non-target membranes protected from lysis? How many monomers are involved in complex formation, and, given the fact that some apicomplexan PLPs are predicted to encode multiple MACPF domains, are less of these monomers necessary to form a pore? Furthermore, the role of the unconserved N-terminal regions of apicomplexan PLPs, which vary in length and sequence, remains to be elucidated. Interestingly, some processes, such as the traversal of epithelial cells of the mosquito midgut by *Plasmodium* ookinetes involve several different PLPs. The interplay of these proteins and potential co-dependencies will be the focus of further studies.

## Author Contributions

JS, GP, and SB wrote the manuscript. All authors contributed to the manuscript and approved the submitted version.

## Conflict of Interest

The authors declare that the research was conducted in the absence of any commercial or financial relationships that could be construed as a potential conflict of interest.
